# Associations of plasma HDL subclasses with incident dementia risk in older adults

**DOI:** 10.1002/alz.71893

**Published:** 2026-09-30

**Authors:** Binchong An, Suad Ali, Karen Smith, Samantha Roemhild, Lindsey Neumann, Dwight R. Stoll, Ling Li, Pamela L Lutsey, Michelle M Mielke, Fang Yu, Ron C Hoogeveen, Lin Zhang, Alvaro Alonso, Danni Li

**Affiliations:** ^1^ Department of Lab Medicine and Pathology University of Minnesota Minneapolis Minnesota USA; ^2^ Department of Psychiatry University of Wisconsin Madison Wisconsin USA; ^3^ Department of Chemistry Gustavus Adolphus College St Peter Minnesota USA; ^4^ Department of Experimental and Clinical Pharmacology University of Minnesota Minneapolis Minnesota USA; ^5^ School of Public Health University of Minnesota Minneapolis Minnesota USA; ^6^ Department of Epidemiology and Prevention Wake Forest University School of Medicine Winston‐Salem North Carolina USA; ^7^ Edson College of Nursing and Health Innovation Arizona State University Phoenix Arizona USA; ^8^ Division of Cardiovascular Research Department of Medicine Baylor College of Medicine Houston Texas USA; ^9^ Division of Biostatistics and Health Data Science School of Public Health University of Minnesota Minneapolis Minnesota USA; ^10^ Department of Epidemiology Rollins School of Public Health Emory University Atlanta Georgia USA

**Keywords:** ARIC, Atherosclerosis Risk in Communities, HDL, high‐density lipoprotein subclasses, incident dementia risk, lipid composition, NMR‐derived small HDL, NMR‐derived very large HDL, UK Biobank, UKB

## Abstract

**INTRODUCTION:**

Plasma high‐density lipoprotein (HDL) varies in size. We examined the associations between HDL subclasses and their lipid composition and incident dementia risk in older adults.

**METHODS:**

We included 23,790 United Kingdom (UK) Biobank (UKB) and 147 Atherosclerosis Risk in Communities (ARIC) older adults free of dementia at baseline. HDL subclasses and their lipid composition were characterized using two distinct approaches: Nightingale nuclear magnetic resonance (NMR) spectroscopy in UKB and anion‐exchange chromatography followed by mass spectrometry‐based lipidomics of physically isolated HDL subfractions (AEX‐MS) in ARIC. Cox regression models were used to evaluate the associations, adjusting for covariates.

**RESULTS:**

In the UKB, Nightingale NMR‐derived plasma small and very large HDL particle and six lipid concentrations (total lipids, triglycerides [TG], phospholipids, free cholesterol, cholesterol ester [CE], total cholesterol) were associated with lower and higher risks of incident dementia, respectively. These associations were likely driven mainly by particle concentration in NMR‐derived small HDL and by both lipid and lipid concentrations in NMR‐derived very large HDL. In the ARIC, total TG in AEX‐defined HDL subfraction 1 (e.g., smaller HDL), total lysophosphatidylcholine (LPC) and phosphatidylethanolamine (PE), and TG in AEX‐defined subfraction 2 (e.g., larger HDL) were associated with a lower incident dementia risk.

**DISCUSSION:**

Although different HDL subclass characterization and lipid measurement methods made it difficult to compare results across cohorts, HDL subclasses and their lipid compositions were associated with the risk of incident dementia in both cohorts.

## Highlights

- HDL subclasses were differentially associated with risk of incident dementia.
- NMR‐derived small HDL was associated with lower risk of incident dementia.
- NMR‐derived very large HDL was associated with higher risk of incident dementia.

## 1 BACKGROUND

Plasma high‐density lipoprotein (HDL) comprises lipoproteins of varying sizes. Emerging evidence suggests that plasma HDL’s association with incident dementia may be size‐dependent. Small HDL, known for its enhanced cholesterol efflux capacity and vascular‐protective effects through multiple pathways,^1^, ^2^, ^3^, ^4^ is reduced in older adults with AD dementia.^5^, ^6^ Small HDL can enter the central nervous system (CNS), help reduce CNS inflammation, and facilitate lipid clearance from the CNS to the periphery.^6^ In contrast, very elevated levels of HDL cholesterol (HDL‐C), presumably reflective of dysfunctional very large HDL, increase the risk of dementia.^7^, ^8^ Furthermore, over 200 individual lipid species have been identified in plasma HDL.^9^ HDL‐C, the sum of free cholesterol and cholesterol esters on HDL, is associated with a lower risk of dementia.^10^, ^11^, ^12^ However, some reports have shown that high HDL‐C is associated with a higher risk of dementia.^7^, ^8^ Beyond HDL‐C, the roles of other lipid species or classes in HDL, and their associations with incident dementia risk, remain unknown.

We hypothesize that plasma HDL size‐dependent subclasses (i.e., smaller and larger HDL) are differentially associated with the risk of incident dementia. To this end, we examine associations of HDL size‐dependent subclasses and their lipid composition with incident dementia risk using the longitudinal UK Biobank (UKB) and Atherosclerosis Risk in Communities (ARIC) cohorts. Baseline HDL subclasses and their lipid composition in these two cohorts are assessed using different approaches: Nightingale nuclear magnetic resonance (NMR) spectroscopy in the UKB cohort and mass spectrometry‐based lipidomics of HDL subfractions physically separated by anion‐exchange chromatography (AEX‐MS) in the ARIC cohort. The Nightingale NMR does not physically separate HDL subclasses and it estimates particle and lipid concentrations via signal deconvolution, whereas the AEX‐MS method physically separates HDL subclasses and provides measurements of a broader range of lipid classes and individual lipid species within HDL subclasses. The UKB cohort has a much larger sample size than the ARIC cohort and is the primary test of the hypothesis, whereas the ARIC cohort is for hypothesis generation.  In the UKB cohort, HDL has four size‐dependent subclasses, designated as small (S), medium (M), large (L), and very large (VL), with average sizes of 8.7, 10.9, 12.1, and 14.3 nm, respectively.^13^ In the ARIC cohort, HDL is separated by AEX into HDL subfractions 1 and 2, with subfraction 1 presumably smaller than subfraction 2.

## 2 METHODS

### 2.1 Study cohorts

UKB is a large prospective cohort of 502,128 participants aged 39 to 73 years, recruited from 22 centers across the United Kingdom (2006 to 2010). Baseline assessments included questionnaires, physical measurements, and the collection of blood, urine, and saliva samples. A subset of 292,000 participants underwent NMR‐based metabolite profiling (Nightingale Health). For this analysis, participants aged 67 years or older were included to align with the ARIC cohort. Participant selection is shown in Figure S1. The Northwest Multi‐Center Research Ethics Committee approved the UKB study, and all participants provided written informed consent.

The ARIC study is a prospective cohort of 15,792 adults aged 45 to 64 years recruited from four US communities (1987 to 1989), with follow‐up through in‐person visits, telephone contacts, hospitalization surveillance, and death record linkage. This analysis used Visit 5 (2011 to 2013) as the baseline. Eligible participants had fasting plasma samples available, were free of dementia at baseline, and had brain MRI data. Of 1721 eligible participants, a larger sample was initially selected at random; however, due to freezer‐related sample loss, 147 participants were included in the final analysis, and thus the analytic sample was not strictly random. Institutional Review Boards approved the ARIC study at all participating institutions, and participants provided written informed consent.

### 2.2 NMR‐based plasma metabolite profiling in UKB

In the UKB, plasma samples from 292,000 participants were analyzed using Nightingale NMR metabolite profiling following detailed protocols,^14^, ^15^, ^16^, ^17^ which include four HDL subclasses, each with particle and six lipid concentrations (total lipids, triglycerides [TG], phospholipids, free cholesterol, cholesterol ester [CE], and total cholesterol) (Table S1).

**RESEARCH IN CONTEXT**

1. **Systematic review**: In general, higher plasma HDL‐C is associated with a lower risk of incident dementia, although some reports have shown that high HDL‐C is associated with a higher risk. HDL‐C does not reflect the functionality of HDL. Plasma HDL varies in size, lipid composition, and ability to protect vascular integrity. In this study, we investigated the associations between HDL size‐dependent subclasses and their lipid composition, as well as the risk of incident dementia, in two UKB and ARIC cohorts.
2. **Interpretation**: HDL subclasses and their lipid composition were associated with the risk of incident dementia in both cohorts. In the UKB, we observed that NMR‐derived small HDL and very large HDL particle and lipid concentrations were associated with lower and higher risks of incident dementia, respectively, among UKB participants aged 67 years or older, consistent with our hypothesis that HDL subclasses are differentially associated with the risk of incident dementia. In the ARIC cohort, total TG in the anion‐exchange chromatography (AEX)‐defined HDL subfraction 1 (e.g., smaller HDL) and total LPC, PE, and TG in the AEX‐defined HDL subfraction 2 (e.g., larger HDL) were associated with a lower risk of incident dementia. However, we acknowledge that key differences between these cohorts made direct comparisons difficult.
3. **Future directions**: UKB findings, despite the larger sample size, require replication in an independent cohort. Given the small sample sizes in ARIC, larger AEX‐MS lipidomic studies with dementia follow‐up are needed. Future work is needed to define the size of AEX‐defined HDL subclasses.

### 2.3 AEX‐MS lipidomic analysis of HDL subfractions in the ARIC

HDL subfractions 1 and 2 were separated using an AEX method^18^, ^19^, ^20^ (Figure S2A). These AEX‐defined subfractions are not directly comparable to conventionally defined HDL subclasses in the literature (i.e., HDL2 and HDL3). Biochemical measurement of proteins and lipids in AEX‐defined HDL subfractions 1 and 2 confirmed that HDL subfraction 1 was smaller than HDL subfraction 2 (Figure S2B–F). Details of biochemical measurements, plasma sample preparation, AEX separation of plasma lipoproteins, lipid extraction protocol, and mass spectrometry analysis are described in the supporting information. Briefly, 200 µL of a prepared plasma sample (1:1 diluted using PBS and filtered) was injected into an AEX column, which separated HDL into subfractions 1 and 2, defined by respective elution windows. Collection of eluents from these windows resulted in 0.88mL and 2.32 mL of HDL subfractions 1 and 2, respectively. For lipid extraction, 100 µL of eluent from each AEX‐defined HDL subfraction, along with 20 µL of internal standards, was extracted using a butanol‐methanol lipid extraction protocol. The resulting lipid extract was dried down and resuspended in 50 µL of buffer composed of HPLC‐grade chloroform:methanol (1:1) containing 100 µg/mL butylated hydroxytoluene (BTH).

Furthermore, 2 µL of resuspended lipids were directly infused into a QQQ mass spectrometer at a flow rate of 100 µL/min using a running buffer made of 10 mM ammonium formate, and isopropyl alcohol:methanol:chloroform in a 4:2:1 ratio. Selected reaction monitoring transitions were used to detect 249 lipid species belonging to seven lipid classes (Table S2A). Peak areas for each lipid species and internal standard were extracted using Skyline, and peak quality was manually inspected. The relative abundance of each lipid species was calculated as the ratio of the peak area of the lipid species to the peak area of its corresponding internal standard. Quality control samples were used to calculate coefficient of variation (CV). Only lipids with CV ≤ 30% were included in subsequent analyses to ensure acceptable measurement precision (Table S2B). Table S2C lists the number of lipid species included under each lipid class.

### 2.4 Incident Dementia

In the UKB, dementia was ascertained using algorithmically defined outcomes based on self‐report, hospitalizations, and death records.^21^ Follow‐up was calculated from baseline to the first occurrence of incident dementia, death, or administrative censoring (March 31, 2023 for England; August 31, 2022 for Scotland; and May 31, 2022 for Wales), whichever occurred first.

In ARIC, dementia was identified through in‐person neuropsychological assessments (Visit 5 to 9), hospitalization and death records, and telephone follow‐up with participants or informants. ^22^, ^23^ The onset date was defined as the earliest identification across sources.^24^ Participants were followed from baseline until incident dementia, death, loss to follow‐up, or administrative censoring, with censoring at the last contact or administrative date.

### 2.5 Statistical analysis

Cox proportional hazards models were used to assess associations between HDL size‐dependent subclasses and their lipid composition and incident dementia risk, adjusting for covariates. Detailed descriptions of covariates used in the UKB and in ARIC cohorts are provided in the supporting information. In the UKB cohort analysis, each NMR‐derived HDL subclass particle or lipid concentration was log‐ and z‐transformed, and the analysis was performed on complete‐case data. Multiple comparisons were addressed using Benjamini‐Hochberg (BH)‐adjusted *p* values (significance threshold: < 0.05) across the 28 lipid measures, including particle concentration and six lipid class concentrations for each of four HDL subclasses or 24 lipid measures in the case of lipid content per particle analysis. Interactions with sex, *APOE* ε4 carrier status (yes or no presence of the ε4 allele), or lipid‐lowering therapy (LLT) use (females, non‐*APOE* ε4 carriers, and participants not receiving LLT served as the respective reference groups) were assessed using multiplicative terms and considered significant at BH–adjusted *p* < 0.05 across the 28 lipid measures. Subgroup analyses were conducted by use of LLT (yes or no). Landmark/lag and Fine–Gray sensitivity analyses were conducted to evaluate the impact of reverse causation and competing mortality risk, respectively. NMR‐derived plasma TG and total cholesterol were the sums across all 14 NMR‐derived plasma lipoprotein subclasses in the Nightingale NMR profile, and NMR‐derived plasma HDL‐C was the aggregated total cholesterol across the four NMR‐derived HDL subclasses.

In the ARIC cohort analysis, we assessed the associations of total lipid per lipid class and individual lipid species in AEX‐defined HDL subfractions 1 and 2 with incident dementia risk. Total lipid abundance for each lipid class was primarily defined as the mean of the log2‐transformed lipid ratios across all individual lipid species within that class. As an alternative definition, we also calculated total lipid abundance as the sum of the log2‐transformed lipid ratios across all species within each class. Seven lipid classes (CE, LPC, phosphatidylcholine [PC], PE, phosphatidylinositol [PI], sphingomyelin [SM], TG) were included in the total lipid analysis. Individual lipid species were also log2‐transformed. Unlike in the UKB cohort, no additional Z‐transformation was applied to the log2‐transformed total lipid measures or individual lipid species. We used a minimal set of covariates for adjustment: age, sex, center, *APOE* ε4 carrier status (yes or no), cognitive impairment status (normal cognition or mild cognitive impairment), use of LLT (yes or no), and smoking status (never or ever smoker). Given the modest size of the ARIC cohort (*n* = 147) and the limited number of incident dementia cases (*n* = 38) relative to the number of covariates included in the multivariable Cox proportional hazards models, standard maximum partial likelihood estimation was expected to be susceptible to small‐sample bias, including inflated or unstable standard errors and attenuated hazard ratio (HR) estimates under sparse‐data conditions. To address this, Firth's penalized partial likelihood approach^25^ was applied as the primary analytic method. For comparison and transparency, corresponding unpenalized (standard) Cox models were also fit and reported.

Because the analyses in the ARIC cohort were intended to be hypothesis‐generating, we report both *p* and BH‐adjusted *p* and interpret the findings with appropriate caution. For total lipid analysis, BH‐adjusted *p* was applied across seven total lipid measures in either AEX‐defined HDL subfraction; for individual lipid analysis, BH‐adjusted *p* was applied across 152 individual lipid measures in AEX‐defined HDL subfraction 1 and 137 in AEX‐defined subfraction 2. Missingness was low across our covariates. To address covariate missingness, we used multiple imputation by chained equations, generating 10 imputed datasets, each with 30 iterations, to ensure convergence. In ARIC, plasma TG, HDL‐C, and total cholesterol were measured using enzymatic assays on automated Roche instruments, independent of the AEX‐MS approach employed in this study.

## 3 RESULTS

### 3.1 Baseline characteristics of the UKB study participants

Among the 23,787 UKB participants included in this study, the average (SD) age at baseline was 68.0 (0.9) years, with 51% female and 97% White. The average (SD) follow‐up period was 12.6 (2.8) years, during which 1626 (6.8%) developed dementia. Detailed baseline characteristics of the UKB study participants are shown in Table 1.

**TABLE 1:** Baseline characteristics of UKB study participants.

Characteristics	Total 23,787	Non‐Dementia 22,161	Dementia 1626	*p* value
Age (years)	68.0 (0.9)	68.0 (0.9)	68.2 (0.9)	<0.001
Gender				0.006
Male (*n* [%])	11,630 (49%)	10,782 (49%)	848 (52%)	
Female (*n* [%])	12,157 (51%)	11,379 (51%)	778 (48%)	
Ever smoked				0.84
No (*n* [%])	8604 (36%)	8012 (36%)	592 (36%)	
Yes (*n* [%])	15,183 (64%)	14,149 (64%)	1034 (64%)	
BMI	27.5 (4.3)	27.5 (4.3)	27.6 (4.5)	0.71
Anti‐hypertension medication use				0.007
No (*n* [%])	19,648 (83%)	18,345 (83%)	1,303 (80%)	
Yes (*n* [%])	4139 (17%)	3816 (17%)	323 (20%)	
Lipid‐lowering therapy use				0.004
No (*n* [%])	20,462 (86%)	19,102 (86%)	1360 (84%)	
Yes (*n* [%])	3325 (14%)	3059 (14%)	266 (16%)	
Diabetes				<0.001
No (*n* [%])	21,816 (92%)	20,402 (92%)	1414 (87%)	
Yes (*n* [%])	1971 (8%)	1759 (8%)	212 (13%)	
Systolic blood pressure	146.4 (19.0)	146.4 (18.9)	146.2 (19.3)	0.81
Education				0.007
No (*n* [%])	18,486 (78%)	17,179 (78%)	1307 (80%)	
Yes (*n* [%])	5301 (22%)	4982 (22%)	319 (20%)	
Race				0.47
Non‐White (*n* [%])	706 (3%)	653 (3%)	53 (3%)	
White (*n* [%])	23,081 (97%)	21,508 (97%)	1573 (97%)	
APOE ε4 carrier status				<0.001
No (*n* [%])	17,150 (72%)	16,417 (74%)	733 (45%)	
Yes (*n* [%])	6637 (28%)	5744 (26%)	893 (55%)	
Time to event (years)	12.6 (2.8)	12.8 (2.7)	10.0 (3.1)	<0.001

*Note*: Continuous variables were compared between groups using the Wilcoxon rank‐sum test, and categorical variables were compared using Pearson's chi‐squared test.

### 3.2 Association of NMR‐derived HDL subclass particle or lipid concentration with incident dementia in UKB cohort

All six NMR‐derived small HDL lipid (total lipids, TG, phospholipids, free cholesterol, CE, total cholesterol) and particle concentrations were associated with a lower risk of incident dementia in Model 1, which adjusted for age, sex, race, *APOE* ε4 carrier status, education, body mass index, smoking, diabetes, systolic blood pressure, antihypertensive medication use, and LLT use (HRs: 0.89 to 0.91, BH‐adjusted *p* range: ≤0.001) (Figure 1A and Table S3A1). NMR‐derived medium HDL TG concentration was associated with a lower risk of incident dementia in Model 1 (HR: 0.92 [0.88 to 0.97], BH‐adjusted *p* = 0.003) (Figure 1B). None of the NMR‐derived large HDL lipid or particle concentrations were associated with the risk of incident dementia in Model 1 (Figure 1C). The five NMR‐derived very large HDL lipid (total lipids, phospholipids, free cholesterol, CE, and total cholesterol), except TG, and particle concentrations were associated with a higher risk of incident dementia (HRs: 1.10 to 1.14, BH‐adjusted *p* range: < 0.001 to 0.015) in Model 1 (Figure 1D). We conducted landmark sensitivity analyses excluding incident dementia cases occurring within the first 2, 3, or 5 years of follow‐up to minimize potential reverse causation. The associations remained materially unchanged (Table S3B). We also conducted Fine–Gray competing‐risk analyses that accounted for death as a competing event. The associations also remained largely unchanged (Table S3C).

**FIGURE 1:**
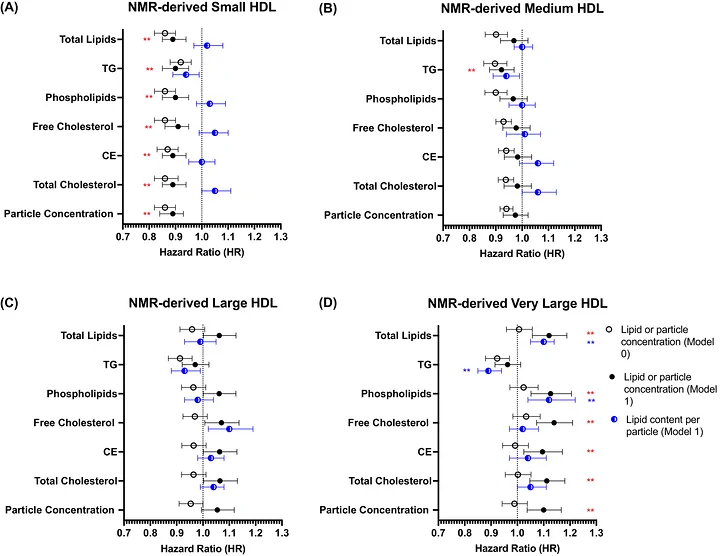
Associations between NMR‐derived HDL subclass lipid/particle concentration or lipid content per particle and incident dementia risk in UKB study cohort. Cox regression models were used to evaluate the associations between each log‐ and z‐transformed NMR‐derived HDL subclass lipid/particle concentration and incident dementia risk. Model 0 included no covariates; Model 1 adjusted for age, sex, race, *APOE* ε4 carrier status, education, BMI, smoking, diabetes, SBP, antihypertensive medication use, and LLT use. Lipid content per particle was calculated using lipid concentration divided by particle concentration of the respective HDL subclass, and the ratio was log‐ and z‐transformed. Cox regression models were used to evaluate the associations between lipid content per particle and incident dementia risk. The hazard ratios (HRs) represent the relative risk of incident dementia per 1‐unit increase in log‐ or z‐transformed lipid/particle concentration or lipid content per particle. BH‐adjusted *p* values reflect the statistical significance of this association after controlling for multiple testing of 28 HDL subclass lipid/particle concentrations or 24 HDL subclass lipid contents per particle. **BH *p* value < 0.05 in Model 1s.

To assess whether the observed associations between NMR‐derived HDL subclasses and incident dementia reflected lipid composition or particle number, we calculated the ratio of each lipid to particle concentration (i.e., lipid content per particle) as a predictor and repeated the Cox regression analysis. After correction for multiple comparisons of 24 lipid‐to‐particle ratios across four HDL subclasses, none of the lipid content per particle in NMR‐derived small, medium, or large HDL was statistically significantly associated with incident dementia risk (Figure 1A–C). Nevertheless, TG content per particle in NMR‐derived small, medium, or large HDL had HRs of 0.93 to 0.94 with a BH‐adjusted *p* range of 0.073 to 0.079. NMR‐derived very large HDL TG content per particle (HR: 0.89 [0.85 to 0.94], BH‐adjusted *p* = 0.001) was associated with a lower risk, whereas NMR‐derived very large HDL total lipid content per particle (HR: 1.10 [1.05 to 1.14], BH‐adjusted *p*: < 0.001) and phospholipid content per particle (HR: 1.12 [1.04 to 1.22], BH *p* = 0.041) were associated with a higher incident dementia risk (Figure 1D and Table S3A2).

To assess whether associations were confounded by overall plasma lipid burden, we further adjusted Model 1 for NMR‐derived plasma TG, total cholesterol, or HDL‐C. Notably, NMR‐derived plasma TG and total cholesterol were significantly associated with incident dementia risk (HR: 0.90 [0.86 to 0.95], BH‐adjusted *p* < 0.001, and HR: 0.94 [0.89 to 0.99], BH‐adjusted *p* = 0.026, respectively). NMR‐derived total HDL‐C was not significantly associated with incident dementia risk (HR: 1.01 [0.96 to 1.07], BH‐adjusted *p* = 0.632) (Table S3D).

Adjustment for NMR‐derived plasma TG did not significantly change the associations of lipid or particle concentration of NMR‐derived small HDL except for TG (Table S3A1 and Figure S3). Because NMR‐derived small HDL TG and NMR‐derived plasma TG are highly correlated (Pearson correlation coefficient of 0.91; Table S3E), adjusting for NMR‐derived plasma TG caused collinearity (Variance Inflation Factor [VIF] = 5.9) (Table S3F). To further address this issue, we constructed a NMR‐derived small HDL TG‐to‐plasma TG ratio as a predictor, which showed no association with incident dementia (HR:1.04 [0.99 to 1.09]) (Table S3D). Adjustment for NMR‐derived plasma TG attenuated the association of NMR‐derived medium HDL TG as well but did not significantly change the association of other lipid or particle concentrations of NMR‐derived medium HDL, nor did it change the association of lipid or particle concentrations of NMR‐derived large HDL (Table S3A1 and Figure S3). After adjusting for NMR‐derived plasma TG, NMR‐derived very large HDL free cholesterol, phospholipids, and total lipid concentrations remained associated with a higher risk (HRs:1.09 to 1.11), and NMR‐derived very large HDL CE and particle concentration were no longer significant, although particle concentration was still associated with incident dementia, with a HR of 1.07 (1.01 to 1.14) and a BH‐adjusted *p* of 0.055 (Table S3A1 and Figure S3).

Adjustment for NMR‐derived plasma total cholesterol did not significantly change any of the associations of lipid or particle concentrations in NMR‐derived small or medium HDL but strengthened the associations for lipid and particle concentrations in NMR‐derived large and very large HDL, except for TGs (Table S3A1 and Figure S4).

Associations of NMR‐derived HDL subclasses and their lipid composition with incident dementia risk were sensitive to the adjustment for NMR‐derived plasma HDL‐C. The overall trend was that the adjustment strengthened the associations of each HDL subclass (Table S3A1 and Figure S5). However, given moderate to high collinearity between lipid and particle concentrations of NMR‐derived medium or large HDL and NMR‐derived HDL‐C (VIFs ranged from 3.9 to 7.7) (Table S3F), estimates from models additionally adjusted for HDL‐C should be interpreted with caution.

### 3.3 Interaction between NMR‐derived HDL subclass lipid measures and sex, APOE ε4 carrier status, or LLT use in UKB cohort

None of the NMR‐derived lipid measures from HDL subclasses showed evidence of a statistically significant interaction with sex or *APOE* ε4 carrier status (Tables S3G,H). Interaction with LLT use was significant for NMR‐derived small, medium, and very large HDL TG (BH‐adjusted *p* range: 0.013 to 0.018) (Table S3I). Among UKB participants who were non‐users of LLT, NMR‐derived small, medium, and very large HDL TG were associated with a lower risk of incident dementia (HRs: 0.87 to 0.94), whereas these associations were absent or reversed among LLT users (HRs: 1.07 to 1.15).

### 3.4 Baseline characteristics of the ARIC study participants

The mean (SD) age of the 147 ARIC Visit 5 (baseline) participants without dementia was 75.4 (5.1) years; 61% (90/147) were female, 52% (77/147) were White, and 20% (30/147) had mild cognitive impairment (MCI). The mean (SD) follow‐up was 7.7 (2.8) years, during which 74.1% (109/147) remained dementia‐free, and 25.9% (38/147) developed incident dementia. Detailed baseline characteristics of the ARIC study participants are presented in Table 2. Compared with UKB participants, the ARIC cohort had a higher mean age (75.4 vs 68.0 years), lower proportions of males (39% vs 49%) and White participants (52% vs 97%), and higher percentages of LLT use (52% vs 14%), diabetes (37% vs 8%), and hypertension (77% vs 17%).

**TABLE 2:** Baseline characteristics of ARIC study participants.

Characteristics	Total (147)	Non‐dementia (109)	Dementia (38)	*p* value
Age (years)	75.4 (5.1)	74.4 (4.9)	78.3 (4.5)	<0.001
Gender				0.38
Male (*n* [%])	57 (39%)	40 (37%)	17 (47%)	
Female (*n* [%])	90 (61%)	69 (63%)	21 (55%)	
Education				0.3
Basic education (*n* [%])	20 (14%)	12 (11%)	8 (21%)	
Intermediate education (*n* [%])	65 (44%)	50 (46%)	15 (39.5%)	
Advanced education (*n* [%])	62 (42%)	47 (43%)	15 (39.5%)	
Race				0.064
Non‐White (*n* [%])	70 (48%)	47 (43%)	23 (60.5%)	
White (*n* [%])	77 (52%)	62 (57%)	15 (39.5%)	
Smoking				0.15
Never smokers (*n* [%])	141 (96%)	109 (97%)	32 (91%)	
Ever smokers (*n* [%])	6 (4%)	3 (3%)	3 (9%)	
Lipid‐lowering therapy use				0.08
No (*n* [%])	71 (48%)	48 (44%)	23 (60.5%)	
Yes (*n* [%])	76 (52%)	61 (56%)	15 (39.5%)	
Cognitive status				0.014
CN (*n* [%])	117 (80%)	92 (84%)	25 (66%)	
MCI (*n* [%])	30 (20%)	17 (16%)	13 (34%)	
Diabetes status				0.42
No (*n* [%])	92 (62%)	66 (61%)	26 (68%)	
Yes (*n* [%])	54 (37%)	42 (39%)	12 (32%)	
Missing (*n* [%])	1 (1%)	1 (0%)	0(0%)	
Hypertension status				0.32
No (*n* [%])	34 (23%)	23 (21%)	11 (29%)	
Yes (*n* [%])	113 (77%)	86 (79%)	27 (71%)	
BMI (kg/m^2^)	28.2(4.9)	28.7(4.7)	26.7(5.1)	0.051
APOE ε4 carrier status				0.72
No (*n* [%])	93 (63%)	69 (63%)	24 (63%)	
Yes (*n* [%])	49 (33%)	35 (32%)	14 (37%)	
Missing (*n* [%])	5 (4%)	5 (5%)	0 (0%)	
Time to event (years)	7.7 (2.8)	8.2 (2.7)	6.3 (2.6)	<0.001

*Note*: Continuous variables were compared between groups using the Wilcoxon rank‐sum test, and categorical variables were compared using Pearson's chi‐squared test.

### 3.5 Association of AEX‐defined HDL subfraction total lipid level with incident dementia in ARIC cohort

In Firth's penalized Cox Model 1, higher TG in AEX‐defined HDL subfraction 1 (HR of 0.43 [0.23 to 0.81], *p* = 0.009) and higher LPC, PE, and TG in AEX‐defined HDL subfraction 2 (HRs of 0.37 [0.16, 0.87], 0.50 [0.28, 0.90], 0.51 [0.31, 0.83], *p* = 0.022, 0.021, 0.007, respectively) were associated with lower risk of incident dementia, remaining borderline significant after correction for multiple comparisons of seven lipid classes (BH‐adjusted *p* range of 0.050 to 0.060) (Figure 2 and Table S4A). Model 1s without Firth penalization yielded consistent but attenuated estimates (Figure 2). Further adjustment for plasma TG, HDL‐C, and total cholesterol did not change the associations (Table S4A). Of note, none of plasma TG, total cholesterol, or HDL‐C were associated with incident dementia risk after adjustment for covariates in the 147 ARIC participants included or the entire ARIC Visit 5 cohort (Table S4B). Calculating a total lipid level using the log‐transformed summed lipid ratio across all lipid species yielded similar results (Table S4C).

**FIGURE 2:**
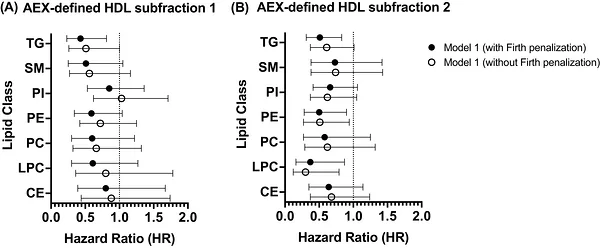
Association of AEX‐defined HDL subfraction 1 or 2 total lipid per lipid class of seven lipid classes and incident dementia in ARIC study cohort. Cox regression models were used to evaluate the associations between each total lipid of a lipid class and incident dementia in AEX‐defined HDL subfraction 1 (A) and HDL subfraction 2 (B). Model 1 was run with or without Firth's penalization, adjusted for age, sex, race, *APOE* ε4 carrier status, cognitive impairment status, statin use, and smoking status. The hazard ratio (HR) represents the relative risk of incident dementia associated with a one‐unit increase in the mean of the log2‐transformed ratios of all lipids within each lipid class.

### 3.6 Association of AEX‐defined HDL subfraction individual lipid species and incident dementia in ARIC cohort

In Firth's penalized Cox Model 1, 25 of the lipid species in AEX‐defined HDL subfraction 1 and 14 lipid species in the AEX‐defined HDL subfraction 2 were associated with a lower risk of incident dementia. Of the 25 lipid species in AEX‐defined HDL subfraction 1, 13 were TGs, and seven were SMs, which were associated with a lower risk of incident dementia (HRs: 0.44 to 0.79 for TGs and 0.45 to 0.54 for SMs; *p* range: < 0.05; BH‐adjusted *p* range: 0.075 to 0.277; Table S5A).  Among the 14 lipid species in AEX‐defined HDL subfraction 2, three were TG, four were PEs, four were LPCs, and three were PCs, which were associated with a lower risk of incident dementia (HRs: 0.54 to 0.58 for TGs, 0.49 to 0.62 for PEs, 0.48 to 0.67 for LPCs, and 0.43 to 0.54 for PCs, *p* range: < 0.05; BH‐adjusted *p* range: 0.276 to 0.334; Table S5A). Further adjustment for plasma TG, HDL‐C, or plasma total cholesterol did not attenuate these associations in either AEX‐defined HDL subfraction 1 or 2 (Table S3A).

## 4 DISCUSSION

We examined the associations between HDL subclasses and their lipid composition and incident dementia risk in the UKB and ARIC cohorts. HDL subclasses and lipid composition were associated with risk of incident dementia in both cohorts. In the UKB, we observed that NMR‐derived small HDL and very large HDL particle and lipid concentrations (total lipids, TG, phospholipids, free cholesterol, CE, total cholesterol) were associated with lower and higher risks of incident dementia, respectively, among UKB participants aged 67 years or older, consistent with our hypothesis that HDL subclasses are differentially associated with the risk of incident dementia. In the ARIC cohort, total TG in AEX‐defined HDL subfraction 1 (e.g., smaller HDL) and total LPC, PE, and TG in AEX‐defined HDL subfraction 2 (e.g., larger HDL) were associated with a lower risk of incident dementia.

Key differences between these cohorts made direct comparisons difficult. First, the AEX‐defined HDL subfractions in ARIC are not directly equivalent to the NMR‐defined small, medium, large, and very large HDL subclasses in UKB. Second, the two cohorts use different lipid measurement principles. The Nightingale estimates lipids assigned to the HDL subclasses. The AEX‐MS method uses targeted mass spectrometry to measure extracted lipid species from physically isolated HDL subclasses via selected reaction monitoring, and the corresponding internal standard for each lipid class was used to determine their relative abundances. To our knowledge, no prior literature directly compared plasma HDL subclasses and their lipid composition from the NMR Nightingale metabolic profiling with those from an orthogonal biochemical method. Finally, the two cohorts had very different populations. For the rest of the discussion, we will focus on the UKB results.

There is a well‐documented paradox in the literature on healthy aging whereby plasma lipids, such as TG, are associated with higher incident dementia risk when measured in midlife but with lower (or null) risk when measured in late life.^26^, ^27^ We conducted landmark/lag analyses to ensure the UKB results were robust to reverse causation over ≤5 years and Fine–Gray competing‐risk analyses to ensure the UKB results were robust to death as a major competing event for dementia during follow‐up. In the UKB cohort, NMR‐derived plasma TG was associated with a lower risk of incident dementia. The attenuation of the NMR‐derived small HDL TG association to the null after adjusting for plasma TG and the null association for the NMR‐derived small‐HDL‐TG‐to‐plasma‐TG ratio suggest that NMR‐derived small HDL TG's association with a lower risk of incident dementia was not independent of plasma TG in the UKB cohort. Frailty and nutritional reserve may be plausible explanations for plasma TG and small HDL TG's association with a lower risk of incident dementia.^27^ Nevertheless, the concentrations of NMR‐derived small HDL particles and other lipids besides TG remained associated with a lower risk of incident dementia upon adjustment for plasma TG, total cholesterol, and HDL‐C.

HDL biogenesis begins with lipid‐poor apolipoprotein A‐1 (apoA‐1), which forms spherical HDL via lipidation by ATP‐binding cassette transporter A1 (ABCA1), followed by esterification by lecithin‐cholesterol acyltransferase (LCAT) and enlargement by ATP‐binding cassette transporter G1 (ABCG1)/scavenger receptor class B type I (SR‐BI) and phospholipid transfer protein (PLTP).^28^, ^29^ Remodeling via Cholesteryl Ester Transfer Protein (CETP) exchanges lipids, enriching HDL particles with TG, which lipases hydrolyze, thereby reducing their size.^30^, ^31^ Overall, small HDL formation is favored by conditions that promote high nascent pre‐β HDL production and/or high CETP/lipase‐driven shrinkage. Furthermore, rising insulin resistance and visceral adiposity, some of which may be age‐related, shift HDL lipid composition toward greater TG content and lower CE content.^32^ With hepatic/endothelial lipase activity, these changes tend to push the HDL size distribution toward smaller HDL particles.^32^, ^33^

Because small HDL is formed through multiple processes, we should be cautious when interpreting what insulin resistance‐ or adiposity‐associated small HDL or TG in small HDL reflects and how it relates to the risk of incident dementia. Without direct assessment of pre‐β HDL production, CETP activity, or lipase activity, we cannot determine whether nascent pre‐β HDL formation, CETP/lipase‐driven shrinkage generating small HDL, or both underlie the pool of small HDL associated with a lower risk of incident dementia. Consistent with this uncertainty, the lipid content per particle analysis results showed that, for NMR‐derived small HDL, their associations with a lower risk of incident dementia were driven predominantly by particle concentration rather than lipid composition, a pattern that does not, on its own, help distinguish the possible origins of small HDL.

The association of small HDL and a lower risk of incident dementia is plausibly explained by vascular or systemic mechanisms. Plasma small HDLs enhance endothelial and vascular integrity through multiple pathways,^2^, ^3^, ^4^ all of which have translated into greater protection against cardiovascular diseases and all‐cause mortality.^34^ Furthermore, a more central mechanism involving direct entry into the CNS and the action of apoA1, the principal HDL apolipoprotein, has biological plausibility rather than being pure speculation.^35^, ^36^, ^37^, ^38^ Lipid‐poor apoA1/HDL is the form that effectively crosses the blood‐brain barrier.^39^ However, it is unclear whether lipid‐poor apoA1/HDL is equivalent to Nightingale NMR‐derived small HDL. Martinez et al. also provided indirect evidence that plasma small HDL might enter the CNS by demonstrating a correlation between plasma and cerebrospinal fluid small HDL particle concentrations measured by ion‐mobility mass spectrometry in older adults.^6^

The formation of very large HDL particles is favored by conditions that promote ABCG1/SR‐BI‐ and PLTP‐mediated enlargement and by low CETP/lipase‐driven shrinkage. In some individuals, age‐related declines in CETP‐mediated exchange and hepatic lipase activity favor the accumulation of large, CE‐enriched, low‐turnover HDL particles.^40^, ^41^ Consistent with the previously discussed growth‐and‐remodeling framework, TG content indicates how actively a very large HDL particle cycles through the CETP/lipase exchange pathway, rather than serving as protective cargo. Under this interpretation, a higher proportion of actively turning‐over, TG‐enriched very large HDL particles could offset, at least statistically, whatever process links accumulation of a CE‐enriched very large HDL pool to dementia risk. This interpretation is broadly consistent with our observations that TG content per particle in NMR‐derived very large HDL was associated with a lower risk of incident dementia; the concentration of NMR‐derived very large HDL particles was associated with a higher risk; and the association between very large HDL particle concentration and higher dementia risk became non‐significant after adjustment for plasma TG. The lipid content per particle analysis results showed that, for NMR‐derived very large HDL, their associations with a higher risk of incident dementia were driven by both particle concentration and lipid composition.

We observed a statistically significant interaction between use of LLT and NMR‐derived small, medium, or very large HDL TG on the risk of incident dementia in the UKB cohort. We speculate that use of LLT alters plasma lipoprotein metabolism, and therefore the association observed in non‐users disappeared among users.

This study has several strengths. First, we included two cohorts that measured HDL size‐based subclasses and lipid composition using orthogonal techniques, both with longitudinal designs and long follow‐up. Although the ARIC sample size is small, the UKB sample size is large, providing sufficient statistical power. Second, although this is not the first study to examine plasma Nightingale NMR metabolites in relation to incident dementia in UKB,^42^, ^43^ ours is a hypothesis‐driven analysis focused specifically on HDL subclasses, incorporating additional analyses beyond prior work, including landmark/lag analyses, Fine–Gray competing‐risk models, lipid‐content‐per‐particle analyses, adjustment for overall plasma lipids, and tests of interaction by sex, *APOE* ε4 carrier status, and use of LLT. Third, AEX‐MS in the ARIC cohort identified associations between less abundant lipids, such as LPC and PE, in HDL subclasses (e.g., AEX‐defined HDL subfraction 2) and incident dementia risk, suggesting that we should not only examine the highly abundant lipid species within HDL subclasses.

This study also has several limitations. First, contextualizing our findings within classical HDL subclass‐size definitions is challenging, particularly for ARIC, where direct average particle‐size information was unavailable at this time for AEX‐defined HDL subfractions 1 and 2. Future work will be needed to provide size definitions of AEX‐defined HDL subclasses, which will enable comparison of HDL subclasses characterized by Nightingale NMR and AEX. Second, we lack subclass‐specific functional characterization of HDL, which poses a challenge in comparing the results reported here to the literature. We assessed the baseline cholesterol efflux capacity of whole‐plasma HDL in 20 ARIC participants who developed incident dementia and 20 who did not, and we found no difference (data not shown). However, this bulk measure would not necessarily detect a functional difference confined to a specific subclass. Third, the 147 ARIC participants analyzed here represent fewer than 10% of the 1721 eligible participants in the parent cohort, raising concerns about selection bias and limiting the broader ARIC cohort's representativeness. Consistent with this, LLT use differed between the overall analyzed sample and those who developed incident dementia (52% vs 39.5%), a pattern that, despite adjustment for LLT use, may reflect broader selection biases. Fourth, CETP activity, hepatic and endothelial lipase activity, and common genetic variants known to influence these processes were not examined in either cohort, so the mechanistic interpretations offered here could not be tested directly, and their relevance may vary across populations with different allele frequencies for these variants.

## CONFLICT OF INTEREST STATEMENT

M.M.M. has served on scientific advisory boards and/or has consulted for Althira, Biogen, Beckman Coulter, Cognito Therapeutics, Eisai, Lilly, Merck, Neurogen Biomarking, Novo Nordisk, Roche, and Siemens Healthineers and has received speaking honoraria from Novo Nordisk and Roche. R.C.H. has received research grants (to his institution) from Denka Seiken and serves as a consultant for Denka Seiken. Other authors declare no competing interests. Author disclosures are available in the Supporting Information.

## CONSENT STATEMENT

The ARIC study was approved by Institutional Review Boards at all participating institutions, and participants provided written informed consent.

## Supporting information



Supporting Information

Supporting Information

Supporting Information
