# Supplementary material for: Associations of plasma HDL subclasses with incident dementia risk in older adults
Source: Alzheimers Dement. 2026 Sep 30;22(10):e71893. doi: 10.1002/alz.71893 (PMC13626148; doi:10.1002/alz.71893)
Supplement: Supplementary file 2 [file ALZ-22-e71893-s001.docx]

**Supporting Information**

**Figure S1.** UK Biobank Participants Flow Chart.

**Figure S2.** Characterization of HDL subfractions 1 and 2 isolated from plasma using the anion-exchange based FPLC method in the ARIC cohort.

**Figure S3.** Association of NMR-derived HDL subclasses and their lipid composition and incident dementia, without (Model 1) and with adjustment for plasma TG (Model 1 + plasma TG) in UKB.

**Figure S4.** Association of NMR-derived HDL subclasses and their lipid composition and incident dementia, without (Model 1) and with adjustment for plasma TG (Model 1 + plasma total cholesterol) in UKB.

**Figure S5.** Association of NMR-derived HDL subclasses and their lipid composition and incident dementia, without (Model 1) and with adjustment for plasma TG (Model 1 + plasma HDL-C) in UKB.

**Supplementary Methods.**

**Figure S1:** UK Biobank Participants Flow Chart.

The flow chart describes the selection of participants from the UK Biobank study for validation analysis. Firstly, participants aged 67 or older were chosen. Then, participants without any records in hospital inpatient, death, or self-reported data were excluded. Further, excluding withdrawn participants, only participants with complete data on metabolites (24,275 participants excluded) and covariates (5,526 participants excluded) were finally selected for validation analyses.

**Figure S2.** Characterization of HDL subfractions 1 and 2 isolated from plasma using the anion-exchange based FPLC method in the ARIC cohort.

(**A**). A representative chromatogram of FPLC separation of plasma lipoproteins including HDL subfractions 1 and 2. Comparison of protein concentration (**B**), total cholesterol mass (**C**), and total cholesterol to protein ratio (**D**) between 10 paired HDL subfractions 1 and 2 (n=10) randomly selected from the 147 ARIC participants. Protein concentration and total cholesterol mass were measured using 50 µL of unconcentrated fractionated HDL subfractions 1 and 2 using a BCA assay and an enzymatic assay, respectively. Further characterization of HDL subfractions 1 and 2 of the entire 147 ARIC samples based on their protein mass measured using their respective peak areas under UV absorbance at 280 nm recorded during FPLC separation (**E**), and total cholesterol ester (CE) based on mass spectrometry analysis (**F**). Area under the curve (AUC) of the UV absorbance at 280 nm peaks represents protein mass in HDL subfractions 1 and 2. 100 µL of unconcentrated, fractionated HDL subfraction 1 or 2 was used for lipid extraction along with internal standards. Dried lipids from HDL subfractions 1 and 2 were reconstituted in 50 µL and 100 µL of a solvent for mass spectrometry analysis. Of note, we used log2-transformed summed lipid ratio for all lipid species within CE that met the precision criterion of less than 30% (based on a quality control sample) as the total CE level. *, ***, **** indicate p value less than 0.05, 0.001, 0.0001, respectively.

**Figure S3.** Association of NMR-derived HDL subclasses and their lipid composition and incident dementia, without (Model 1) and with adjustment for plasma TG (Model 1 + plasma TG) in UKB.

Cox regression models were used to evaluate the associations between each log- and z-transformed NMR-derived HDL subclass lipid and particle concentration and incident dementia, adjusted for age, race, *APOE4* carrier status, education, BMI, smoking, diabetes, SBP, antihypertension medication use, and lipid-lowering medication use (Model 1) or adjusting for all the covariates in Model 1 plus NMR-derived plasma TG. The hazard ratios represent the relative risk of incident dementia per 1-unit increase in log- or z-transformed lipid or particle concentration across different HDL sizes.

**Figure S4.** Association of NMR-derived HDL subclasses and their lipid composition and incident dementia, without (Model 1) and with adjustment for plasma Total Cholesterol (Model 1 + Total Cholesterol) in UKB.

Cox regression models were used to evaluate the associations between each log- and z-transformed NMR-derived HDL subclass lipid and particle concentration and incident dementia, adjusted for age, race, *APOE4* carrier status, education, BMI, smoking, diabetes, SBP, antihypertension medication use, and lipid-lowering medication use (Model 1) or adjusting for all the covariates in Model 1 plus NMR-derived plasma total cholesterol. The hazard ratios represent the relative risk of incident dementia per 1-unit increase in log- or z-transformed lipid or particle concentration across different HDL sizes.

**Figure S5.** Association of NMR-derived HDL subclasses and their lipid composition and incident dementia, without (Model 1) and with adjustment for plasma HDL-C (Model 1 + HDL-C) in UKB.

Cox regression models were used to evaluate the associations between each log- and z-transformed NMR-derived HDL subclass lipid and particle concentration and incident dementia, adjusted for age, race, *APOE4* carrier status, education, BMI, smoking, diabetes, SBP, antihypertension medication use, and lipid-lowering medication use (Model 1) or adjusting for all the covariates in Model 1 plus NMR-derived plasma HDL-C. The hazard ratios represent the relative risk of incident dementia per 1-unit increase in log- or z-transformed lipid or particle concentration across different HDL sizes.

**Supplementary Methods**

***Plasma sample preparation***

500 µL never-thawed plasma samples were removed from the -80 °C freezer approximately 30 minutes before injection during the preceding chromatographic run and thawed at room temperature for 25 minutes. Sample preparation was completed during the final 5 minutes of the prior run. To ensure sufficient recoverable volume and minimize sample loss during filtration, ARIC plasma samples were prepared in duplicate using Spin-X centrifuge filter tubes (Sigma-Aldrich, St. Louis, MO, USA) per sample. Each filter was pre-wetted with 150 µL of 1X PBS and centrifuged at 10,050 x g for 1 minute using an Eppendorf 5424 centrifuge, after which the filtrate was discarded. Plasma (150 µL) was diluted in each Spin-X filter tube by adding 150 µL of 1X PBS, then centrifuged at 10,050 × g for 5 minutes. The filtered plasma from both tubes was then combined to yield approximately 600 µL diluted plasma. The combined sample was aspirated into a 1 mL Luer lock syringe fitted with an injection needle. All air bubbles and excess volume were removed, leaving 200 µL of sample for injection. The injection needle was removed, and the syringe was securely attached to the injection port.

***Separation of HDL subfractions using plasma samples from the ARIC study***

An anion-exchange chromatography Fast Protein Liquid Chromatography (FPLC) method was employed to separate HDL subfractions 1 and 2 based on their surface charge differences. The plasma lipoprotein separation was performed on a Bio-Rad NGC chromatography platform (Bio-Rad, Hercules, CA, USA). The system was equipped with an Opti-Guard guard column (1 mm) (Sigma-Aldrich, St. Louis, MO, USA), a TSKgel DEAE-NPR anion exchange guard column (5 μm particle size, 0.5 cm × 4.6 mm i.d.), and a TSKgel DEAE-NPR anion exchange analytical column (2.5 μm particle size, 3.5 cm × 4.6 mm i.d.) (Tosoh Bioscience; Grove City, OH, USA).

Before the plasma sample injection, the column was equilibrated using Buffer A (50 mM Tris [40 mM Tris HCl and 10mM Tris base] and 1mM EDTA, pH 7.5) and Buffer B (Buffer A supplemented with 1.0 M sodium perchlorate, pH 7.5). Following equilibration, 100 μL of 1X PBS was injected and run as a blank to assess baseline stability. Separation was performed using a step gradient consisting of 5% mobile phase B from 0–5 minutes, 10% B from 5–10 minutes, 12% B from 10–15 minutes, 14% B from 15–20 minutes, 16% B from 20–25 minutes, and 50% B from 25–27 minutes. The column was re-equilibrated at 5% mobile phase B for 10 minutes between injections, resulting in a total run time of 37 minutes per sample. Protein elution was monitored by UV absorbance at 280 nm.

Elution windows of 7.2 -8.3 mins and 8.3 – 11.16 mins were used to collect HDL subfractions 1 and 2, respectively. To minimize sample carryover, the injection needle was cleaned prior to each injection with 1 mL of a 20% ethanol solution, followed by 1 mL of Milli-Q water and 1 mL of 1X PBS. The injection loop was additionally flushed with 1 mL of 1X PBS before a new sample was injected.

***Biochemical characterization of HDL subfractions 1 and 2***

To demonstrate that HDL subfractions 1 and 2 differ in size, 10 paired fractionated HDL subfractions 1 and 2 were randomly selected from 147 ARIC participants. Protein concentration and total cholesterol concentration of these HDL subfractions 1 and 2 were measured using a Bicinchoninic acid (BCA) protein assay (Thermo Scientific, Catalog #23227) and an enzymatic assay (Abcam, Catalog# ab65359), respectively. Based on these 10 representative samples, HDL subfraction 1 had a higher protein concentration, a lower total cholesterol concentration, and a lower total cholesterol-to-protein ratio than HDL subfraction 2 (**Figure S2B-D**). Protein mass differences between HDL subfractions 1 and 2 for all 147 paired samples were further compared using their respective UV absorbance peak areas at 280 nm recorded during FPLC separation, and CE differences for all 147 paired HDL subfractions were further compared using lipidomic mass spectrometry data (**Figure S2E-F**). These comparisons further confirmed that HDL subfraction 1 was more enriched in proteins and less in lipids than HDL subfraction 2 and, therefore, was smaller HDL than HDL subfraction 2.

***In-house-made 13-lipid internal standard mixture***

| **Compound Group** | **Compound Name** | **Commercial Source** | **Target Concentration (μM)** |
| --- | --- | --- | --- |
| Cer | Cer(d18:1-d7/18:0) (IS) | Avanti Cat#860677P | 5 |
| dhCer | dhCer(d18:0/ 8:0 (IS) | Avanti Cat#860626P | 5 |
| FFA | FA(18:1) d9 (IS) | Avanti Cat#861809 | 20 |
| Hex2Cer | Hex2Cer(d18:1/15:0) d7 (IS) | Avanti Cat#330727 | 5 |
| HexCer | HexCer(d18:1/15:0) d7 (IS) | Avanti Cat#330729 | 5 |
| LPI | LPI 13:0 (IS) | Avanti Cat#850101P | 2 |
| S1P | S1P(d18:1) d7 (IS) | Avanti Cat#860659 | 4 |
| SpH | Sph(d17:1) (IS) | Avanti Cat#860640P | 2 |
| SM | SM(d18:1/15:0) d9 (IS) | Avanti Cat#860686 | 10 |
| SHexCer | SHexCer(d18:1/12:0) | Avanti Cat#860573P | 4 |
| AC | AC (16:0) d3 | Larodan Cat#71-1746-5 | 1 |
| Cholic acid d4 | Cholic acid d4 | Cayman Cat#20849 | 5 |
| Hex3Cer | Hex3Cer(d18:1/17:0) | Cayman Cat#24876 | 5 |

***Lipid extraction of HDL subfractions 1 and 2***

100μL of HDL subfraction 1 or 2 and 20 μL internal standards (10 µL Avanti Splash Lipidomix [Catalog #330707, Avanti Research, Alabama, USA] and 10 µL in-house-made 13-lipid internal standard mixture [please see the internal standards and their concentrations in the table above]) were added to each glass vial, followed by 300 µL of butanol: methanol (3:1). Samples were mixed for 10 minutes on a Hula mixer. The vial caps were removed and arranged to ensure the caps were placed back on the corresponding vials. Next, 150 µL heptane: ethyl acetate (3:1) was added and mixed for another 10 minutes, followed by a second addition 150 µL heptane: ethyl acetate (3:1) and an additional 10 minutes of mixing. Phase separation was induced by adding 300 µL of 1% acetic acid and mixing for 10 minutes. The upper organic layer (1^st^), totaling 360 µL, was collected into a clean 1 mL glass vial. The remaining aqueous phase was extracted once more time with 320 µL heptane: ethyl acetate (3:1), mixed for 10 minutes, and centrifuged at 37,000 x g for 1 minute when needed. The 2^nd^ upper layer, totaling 320 µL, was transferred to a clean 1 mL glass vial for combination with the 1^st^ upper organic layer. A final extraction (3^rd^) was performed by adding 200 µL of heptane: ethyl acetate (3:1), mixing for 10 minutes, and centrifuging at 37,000 × g for 1 minute, as needed. The 3^rd^ upper layer, totaling 200 µL, was collected into two 100 µL aliquots, yielding a final combined organic extract volume of 880 µL per sample. The extracted samples were dried in the chemical hood using streams of nitrogen gas flowing through a 24-needle gas manifold. Mass spectrometry vials were positioned in an aluminum 24-hole vial rack, and the gas manifold height on the ring stand was adjusted so that the needle tips were placed inside the vials without contacting the solvent. The nitrogen tank valve is opened, and the pressure regulator was adjusted to prevent splashing. Samples were dried under nitrogen for approximately 1 hour, and at the 30-minute mark, the manifold was moved down. If complete drying was not achieved, the vials were kept under nitrogen for an extended period and can be stored at -80 °C. Following evaporation, dried lip extracts were suspended in HPLC-grade chloroform: methanol (1:1) containing 100 µg/mL BHT by adding 50 µL to each vial, vortexing for 3 seconds, allowing 5 minutes for the solution to settle, and transferring the extract into mass spectrometry vials containing 300 µL glass inserts for autosampler loading. Samples were stored at -80 °C until mass spectrometry analysis.

***Direct infusion MS/MS lipid analysis***

A Thermo TSQ Quantiva Triple Quadrupole mass spectrometer was calibrated before each MS analysis of lipid samples. 2 μL of resuspended lipids were directly infused into the mass spectrometer at a flow rate of 100 μl/min using a running buffer made of 10 mM ammonium formate, and isopropyl alcohol: methanol: chloroform in a 4:2:1 ratio. Selective Reaction Monitoring transitions were used to detect 249 lipid species belonging to 7 lipid classes (**Table S1**). Reporting of lipid species followed the published guidelines.^1^ For example, TG (52:5) [NL-18:3] denotes a triglyceride with 52 total carbons and 5 double bonds, identified by a neutral loss corresponding to fatty acid 18:3.

Peak areas for each lipid species and internal standard were extracted using Skyline, and peak quality was manually inspected. Relative abundance of each lipid species was calculated as the ratio of the peak area of the lipid species to the peak area of its respective internal standard. Quality control samples were used to calculate coefficients of variation (CV). Only lipids with CV ≤ 30% were included in subsequent analyses to ensure acceptable measurement precision (**Table S1**). Further, we used the mean log-transformed lipid ratio across all lipid species within each lipid class as the total lipids for that class. TG (52:5) [NL-18:3] denotes a triglyceride with 52 total carbons and 5 double bonds, identified by a neutral loss corresponding to fatty acid 18:3.

***Other Covariates***

In the ARIC cohort, age, sex, race, body mass index (BMI), smoking, education, diabetes, hypertension, statin use, *APOE4* status, and cognitive status were included as covariates. ARIC participants self-reported sex, race, and their education level at the study baseline, and provided self-reported information on smoking and medication at all study visits. BMI was calculated as weight in kilograms divided by height in meters squared, with measurements obtained while participants were wearing light clothing. Diabetes was defined as fasting glucose ≥126 mg/dL, or non-fasting glucose ≥200 mg/dL, or self-reported physician diagnosis of diabetes, or the use of antidiabetic medication. Hypertension was defined as SBP (systolic blood pressure) ≥140 mm Hg or DBP (diastolic blood pressure) ≥ 90 mm Hg, or as the use of high blood pressure medication. Statin medication use is defined as statin use in the past 4 weeks. *APOE4* genotype was determined using the TaqMan assay (Applied Biosystems, Foster City, CA). Cognitive status was adjudicated with details previously reported.^2^

In the UK Biobank cohort, age at recruitment in years was calculated from participants’ date of birth and the date of the initial assessment center visit. Sex was captured from NHS records or self-reported data at recruitment. BMI was derived from height and weight measurements at the initial Assessment Center visit. Ethnicity, smoking status, education, and antihypertension medication/lipid-lowering medication were captured from ACE touchscreen questionnaires. Ethnicity was categorized into white and non-white groups. Education was classified into two categories: those who had achieved a college or university degree and those who had not. Diabetes was obtained from the self-reported medical conditions collected through verbal interviews conducted by trained nurses. Systolic blood pressure was calculated as the average of two automated Omron measurements. *APOE4* carrier status (yes/no) was based on two directly genotyped SNPs (Single-nucleotide polymorphisms): rs429358 and rs7412 on the UK Biobank arrays.^3^

**References**

1.	Liebisch G, Vizcaíno JA, Köfeler H, et al. Shorthand notation for lipid structures derived from mass spectrometry. *Journal of lipid research*. 2013;54(6):1523-1530.

2.	Knopman DS, Gottesman RF, Sharrett AR, et al. Mild cognitive impairment and dementia prevalence: the atherosclerosis risk in communities neurocognitive study. *Alzheimer's & dementia : the journal of the Alzheimer's Association*. 2016;2:1-11.

3.	Bycroft C, Freeman C, Petkova D, et al. The UK Biobank resource with deep phenotyping and genomic data. *Nature*. 2018;562(7726):203-209.
